# Challenges and Pitfalls for Implementing Digital Health Solutions in Clinical Studies in Europe

**DOI:** 10.3389/fdgth.2021.730680

**Published:** 2021-09-28

**Authors:** Marcel Meyerheim, Anna Burns-Gebhart, Kasra Mirzaie, Tina Garani-Papadatos, Yvonne Braun, Norbert Graf

**Affiliations:** ^1^Clinic of Pediatric Oncology and Hematology, Faculty of Medicine, Saarland University Hospital and Saarland University, Homburg, Germany; ^2^Clinic for Pediatric Oncology and Hematology, Hannover Medical School, Hannover, Germany; ^3^Department of Public Health Policy, School of Public Health, University of West Attica, Athens, Greece

**Keywords:** digital health platform, e-health, digital health, digital technology, health care

## Abstract

The increasing number of digital solutions developed for use in clinical health care settings is accompanied by new challenges to develop and conduct clinical studies that include eHealth technologies. Clinical study implementation plans often disregard or underestimate the necessity of additional administrative and logistic tasks required at clinical sites as well as ethical aspects to test digital solutions. Experiences made in the run-up of an observational clinical feasibility study at three international clinical sites in the framework of the MyPal project (https://mypal-project.eu/) result in recommendations to avoid delays and barriers in the planning of such prospective studies in clinical and also palliative care for increased efficiency.

## Introduction

The current era of rapid technological progress and its necessity in the medical field drives the development of innovative eHealth technology. Application areas in medical care span from personalized care (1), oncology (2–4) and palliative care (5, 6) to mental health (7–9). Many national public health institutions have already acknowledged the urgency to adapt to a global imminent digitalization trend (10, 11). The COVID-19 pandemic emphasized the importance of digital health solutions (DHS) (12–14). To determine strengths and limitations of eHealth interventions (3, 15), as well as to assess usability and evaluate the effectiveness of such technologies in clinical research (16–18), recommendations and guidelines for conducting such studies are becoming even more important. Moreover, eHealth and the use of artificial intelligence in medicine pose new technical and ethical challenges, which need to be discussed (19).

The conduct of clinical feasibility studies is an essential prerequisite to check the integrability of DHS into daily clinical routine, to contribute to the evidence base as well as to evaluate effectiveness and usability. This type of studies helps to become aware of problems that could occur during development and implementation (18). Simultaneously, these studies entail new challenges that often become already apparent in the preliminary phase of the respective studies at the clinical sites. These include additional administrative and logistical burden to apply for ethical approval, to set up the necessary technical infrastructure, the overall data management and study documentation, as well as to familiarize and training of involved clinicians and health care professionals (HCPs) working with the software or applications etc. Ethical aspects in particular must be profoundly considered given that data of vulnerable groups such as patients with serious and severe diseases are digitally collected, processed and possibly transferred. Therefore, they have to be managed in compliance with both national and international regulations, e.g., the General Data Protection Regulation GDPR (EU 2016/679) (20), which in particular considers health-related data as highly sensitive and confidential data being assigned to the special categories of personal data.

Due to the involvement of differently organized institutions and bodies, multicenter studies demand an even higher degree of coordination processes and mechanisms, information exchange and general oversight over conduct and requirements for a successful implementation of the planned study. Such challenges can put a substantial strain on locally already established implementation routines for clinical studies, impede and delay the often tight schedule granted in project plans for setting up clinical studies before patient recruitment can be initiated and can lead to unforeseen additional costs (21).

In this policy brief, we portray experiences and observations made in the course of setting up the currently running observational prospective clinical feasibility MyPal-Child study (MyPal4Kids) (22). Firstly, we provide an overview on the study and its context. Secondly, we present deeper insights into the necessary preparatory work that was required to be able to carry out the study. These are structured around two main aspects: ethical and legal considerations; and administrative tasks and logistics at clinical sites. In each section, we present hands-on recommendations for prospective projects and clinical studies in order to overcome pitfalls and to avoid undesired fallbacks when DHS are incorporated.

## Mypal4Kids

A consortium of various clinical researchers and experts on palliative care as well as software developers from seven European countries joined efforts in the EU-funded project MyPal (Grant Agreement No: 825872) to foster health care for cancer patients by developing digital health platforms based on electronic Patient-Reported Outcome (ePRO). With the help of mobile apps, symptoms and current health states and hence the needs of cancer patients and their caregiver(s) can be communicated and reported to the relevant HCPs on site. The evaluation of the digital health platforms will be the outcome of two carefully designed clinical studies, which were running during the draft of this present publication, with two groups of cancer patients: adults and children in diverse health care settings across Europe (23, 24).

The study involving pediatric cancer patients – MyPal4Kids – focuses on the examination of the usage, usability and acceptance of two mobile apps as part of a digital health platform, one developed for children and adolescents from 6–17 years and one for their legal guardians. These apps combine various functions and advantages of ePRO-based protocols together with gaming aspects in order to enhance motivation and participation among patients. Symptom reporting is embedded in an entertaining underwater-themed game. The gamification of such health care apps is called “serious game” (25). Participating patients can report their condition via an app to their treating HCPs independently of in- or outpatient settings. Acting as a proxy for their child, parents have the opportunity to report their child's health condition via the second app and to provide feedback on quality of life, care satisfaction as well as the impact of the disease on the family in the form of ePRO-based questionnaires. The treating HCPs can check the symptoms reported by the study participants in a web platform. The reported data and information are visualized in tables and as graphs such that developmental trajectories can be identified. Further, the HCPs are able to add observed symptoms or specific treatment details themselves on the platform. A local server is hosted for the study at each clinical site. The necessary backend and database for the apps and the web platform are installed on these servers. This type of data deployment was chosen to assure data protection, e.g. to restrict the access to person-related health data within the premises of the clinical technical infrastructure and to enable data transfer of anonymized data for final study evaluation.

The pediatric oncology departments of the University of Saarland as the coordinating center and Hannover Medical School in Germany as well as the University Hospital Brno in the Czech Republic run MyPal4Kids (22).

## Susceptible Steps Before the Study Initiation

### Ethical and Legal Study Evaluation

Pediatric cancer patients and their families constitute an especially vulnerable group due to the seriousness of the disease and its substantial overall impact on the whole family. The special needs of this group and their relatives must be addressed by the care staff at all times and appropriately looked after, starting with the examination and at diagnosis during active disease treatment and patient follow-up as well as accompanying or sole palliative care. Protecting their dignity and privacy are of highest priority and must always be guaranteed. Thus, regardless of whether the study is pharmaceutical or, as in the case of MyPal4Kids, non-pharmaceutical, the corresponding study protocol needs to be profoundly revised with regard to the aforementioned points and approved by the independent ethics committees (IECs) of all clinical sites involved. Positive evaluations from independent ethic committees verify the solidness of the submitted study design and protocol. The essential premise is that changes and remarks requested by the IECs are considered and the submitted documents are revised accordingly. Besides the study protocol, the submission to the IECs includes all information materials on the study that will be made available to the study participants in the context of a possible recruitment.

Planned clinical studies involving the testing of digital innovations, such as applications or software, may encounter additional requests from the IECs, asking for supplementary information or specific documents. In the case of MyPal4Kids, the ethical committees additionally solicited screenshots of each screen view of every implemented mobile app questionnaire to be provided in the respective national language version. The same applied for the information sheets and informed consent forms which also had to contain comprehensive elucidations on the technical measures planned to prevent unauthorized people from gaining access to sensitive data as well as steps taken to de-identify data. Clarifications for the study participants on the purpose of the data collection, their data protection rights and how to claim them, were applicable in compliance with GDPR. One of the three IECs also demanded a statement by the respective clinical site's Data Protection Officer (DPO) regarding the evaluation of the data protection and security measures undertaken in the study, and the data management plan. The official statement of DPO is a requirement which becomes more and more relevant, reflecting the increasing number of DHS and the legal landscape as shaped by GDPR, and has to be considered in these kinds of studies in which personal data is collected online via digital solutions.

### Actionable Recommendations

If a clinical study is conducted in the framework of a bigger research project, consider setting up a project-internal ethics committee of corresponding expertise, ideally at the project's initiation. This will allow to closely supervise and support all further planning steps of the clinical study with regard to the ethical aspects of protecting the individuals' rights and safety, the frequency and sharing scheme of collected data, and overviewing the respective content in the study protocol, information sheets and informed consent forms. The same applies to the timely consultation of competent DPO to supervise legal-compliance.

Make sure to timely collect all information regarding the necessary submission procedures and requirements of the IECs responsible for each clinical site involved. The applicable international and national laws and regulations can vary widely and depend on the type of clinical study design, especially regarding clinical studies which involve medicinal products (26) and medical devices (27). The design of the digital solution as a medical device implicates stricter requirements and additional costs, e.g., for the certification of software. Create a checklist of documents and materials that need to be translated before the submission. The scheduled meeting dates of the IECs should be investigated in advance.

Depending on the age range of study participants, especially in case of young study participants, it is necessary to draft different versions of age-appropriate information sheets and informed consent or assent forms.

Make sure to check if any other type of clearance is officially required besides the clinical study's ethical evaluation depending on the type of study participants e.g., for pediatric patients.

Make sure in advance of the submission that the study protocol and the development status of the digital innovation have reached a stage where all study material, visualizations, mock-ups, information on data management, etc., to be submitted are at a final stage and no major changes are expected subsequently. Investigate and openly communicate in advance which changes and amendments would require another evaluation by IECs. This important aspect can implicate additional costs and delays if not taken care of appropriately.

In the case of multicenter studies, determine a steering clinical site to make the first submission to the corresponding IEC. After consideration and final approval of the requests for changes, successively submit the revised versions to the remaining IECs while referring to the ethical evaluation obtained. Be aware that all subsequent requests for changes of successive IECs have to be communicated in response to the previous committees. If documents or text passages in the software require new translations due to changes, this might result in additional iterative back loops. The timeframe required for these loops depends on the number and extent of remarked issues and the study personnel available on site. This demands elaborate planning and coordination. Regarding MyPal4Kids, it took 4 months from first submission to final ethical evaluation by the IECs of all three clinical sites.

### Administrative Tasks and Logistics

Further crucial milestones in the preliminaries of clinical studies with DHS concern the planning and implementation of the required technical infrastructure. This includes a multiplicity of decisions on data deployment, data security, data protection and handling of personal health data, data transfer, permission roles and data visibility. These tasks require efficient continuous communication and coordination between the technical developers of the digital health solution to be applied in the study as well as study representatives at clinical sites, their respective information technology and legal departments, and potentially DPOs. In particular, this includes the risk and handling of differences in digital literacy, as well as network connectivity problems.

A combination of access-controlled local and central data deployment had been adopted in MyPal4Kids in order to facilitate permission-based data protection and visibility. Local web servers were set up at each clinical site to collect study data independently from each other and one central server to store all aggregated and anonymized study data for the final evaluation and analysis.

### Actionable Recommendations

Make sure to be aware at all times of the several inter-dependencies when it comes to planning tasks and taking decisions: the technical infrastructure set up usually intersects with the content of the information sheets and informed consent forms. This milestone in planning needs to be addressed and finalized IN ADVANCE of the submissions to the IECs due to the difficulty of retrospective modifications and amendments. The planned technical infrastructure is the foundation to draft and revise the required GDPR-compliant documents, such as: Technical and Organizational Measures to ensure the security of data processing (TOM), Records of Processing Activities and a Data Protection Impact Assessment (DPIA) if required (20).

It is indispensable to schedule and perform a comprehensive rehearsal testing of the digital solution in advance of the actual study in order to test all processes planned for the study. This allows to detect bugs and issues, which may impede or disable usability, and helps to establish an iterative feedback loop. Eventually, the engagement of HCPs is required to evaluate the usability of DHS. Therefore, it is advisable to involve HCPs in all planning and development stages.

In case of multicenter studies and several project partners, make sure to decide at an early time point how responsibilities and tasks in data processing and evaluation will be allocated among project members. Accordingly, contact the competent legal departments and DPO to set up GDPR-compliant Joint Controller Agreements or Data Processing Agreements, depending on the settled responsibilities and rights. This can be part of a general contractual agreement on the responsibilities of the respective pair or group of project partners considering the implementation of the clinical study. One crucial and often time-consuming legal decision while drafting contractual agreements in international multicenter studies is the choice of law and the place of jurisdiction in case of breach of contract. For example, some countries do not accept a place of jurisdiction in another country.

The complexity of setting up technical infrastructure at clinical sites necessitates regular and thorough communication with all experts involved including IT departments, technical developers and clinical study members. Consider following steps when consulting the respective IT departments: servers have to be in place and maintained, operating systems have to be set up and corresponding installations of required software performed including regular updates and backups. Single steps may require confirmation by authorized personnel as well as clarifications about technical specifications for mutual understanding of the operating concept.

Consider individual differences in digital literacy among the involved study personnel and participants at the clinical site. Ensure sufficient training sessions for the personnel. This includes drafting and handing out illustrative and supportive training materials and instructions. A reference guide for HCP can help keep track of the first steps in the study. These should include credential creation steps, installation processes and steps for the app, a feedback system and technical support for the participants. In-app tutorials can be a useful tool to efficiently provide required information.

Make sure that all clinical sites meet the technical requirements to use the proposed digital health solution. HCPs should be well equipped with sufficient electronic devices to be able to use and/or demonstrate the respective application or software. If the digital health solution requires an internet connection, on-site access to Wi-Fi, as well as signal strength and stability, should be verified in advance.

It is recommendable to schedule retrospective revisions of scheduled project deliverables and documents which address aspects such as data management plans, since they are likely to be modified along the development of the digital solution in advance of the preliminary work for studies. If revisions are omitted, references to these publicly official deliverables might hint on already outdated information and lead to extended time for clarifications.

DHS need to consider online and offline solutions for applications due to the possibility of connection problems or missing mobile data.

Be aware of and explore the necessity of sustainable funding to develop, operate, and maintain the digital solution of your project.

## Conclusions

DHS have been investigated in clinical studies for a long time, but not many have been widespread yet due to the challenges of integrating them into everyday clinical practice while ensuring usability and data security (28). Nevertheless, DHS offer immense potential to address patient-centered care needs despite challenges and obstacles for their long-term implementation in clinical routine. We have exposed some challenges that may arise at the early stages of implementation of DHS in clinical studies. We provide a large range of useful actionable recommendations on how to avoid common pitfalls and possible challenges that might lead to delays. In particular, efficient communication and exchange of information among all involved parties is a crucial key for the successful set up and testing of DHS in clinical studies, especially when different organizational entities are involved. It is likely that following these recommendations will transition into less problematic, smoother and efficient processes. This in turn might make the provided DHS more attractive to the already limited clinical resources at hand. The final evaluation and analysis of MyPal4Kids will topic further challenges observed with regard to the time frame after study initiation and respective recommendations as an outcome of the study.

## Author Contributions

MM drafted the first and final version of the manuscript as main author. AB-G, KM, TG-P, YB, and NG contributed to critical revisions of the manuscript. All authors contributed to the article and approved the submitted version.

## Funding

MyPal: Fostering Palliative Care of Adults and Children with Cancer through Advanced Patient Reported Outcome Systems, is funded by the Horizon 2020 Framework Programme of the European Union under Grant Agreement No. 825872.

## Conflict of Interest

The authors declare that the research was conducted in the absence of any commercial or financial relationships that could be construed as a potential conflict of interest.

## Publisher's Note

All claims expressed in this article are solely those of the authors and do not necessarily represent those of their affiliated organizations, or those of the publisher, the editors and the reviewers. Any product that may be evaluated in this article, or claim that may be made by its manufacturer, is not guaranteed or endorsed by the publisher.
